# A Histopathological Journey Through Adamantinomatous Craniopharyngioma: A Case Report

**DOI:** 10.7759/cureus.65917

**Published:** 2024-08-01

**Authors:** Ankit Girepunje, K.M. Hiwale, Anil K Agrawal, Suhit Naseri, Tanisha Paramba

**Affiliations:** 1 Pathology, Jawaharlal Nehru Medical College, Datta Meghe Institute of Higher Education and Research, Wardha, IND; 2 Pharmacology, Jawaharlal Nehru Medical College, Datta Meghe Institute of Higher Education and Research, Wardha, IND

**Keywords:** adamantinomatous craniopharyngioma, tumor, surgical resection, braf, rathke’s pouch

## Abstract

Craniopharyngiomas are rare benign neoplasms of epithelial origin. Usually located in the sellar and suprasellar regions, they typically present with symptoms of mass effect, raised intracranial tension, or endocrinological aberrations. Atypical presentations without these symptoms often delay diagnosis and worsen patient prognostic outcome, while timely diagnosis without these symptoms is essential for patient beneficence. Below, we present a case of an adamantinomatous craniopharyngioma in a 50-year-old female with minimal and non-specific symptoms. Radiographic imagining reported the presence of a cystic lesion in the sellar, suprasellar, and parasellar regions before the surgical excision. The patient was informed and a decision was made to undergo surgical resection of the mass lesion. The postoperative histopathologic study confirmed the neoplasm to be an adamantinomatous craniopharyngioma.

## Introduction

Craniopharyngiomas are benign tumors of the embryological Rathke’s pouch (craniopharyngeal duct). It is a low histological-grade epithelial cell tumor originating in the sellar and suprasellar regions [1,2]. They are rare, boasting an incidence of fewer than two cases per million persons, and accounting for a paltry 5% of all brain tumors [2,3]. They are classified into two types- adamantinomatous and papillary- for their histological appearance. These two types exhibit differing population incidences, with the former showing a bimodal distribution in adults and children while the latter is found mainly in the age group of 40 to 55 [4].

Adamantinomatous tumors are driven by mutations in CTNNB1 (encoding β-catenin) which bolsters its stability. Papillary tumors owe genesis to BRAFV600E mutations [5-7]. Even though they are Grade 1 neoplasms, their essential location and high recurrence rate render them regarded as malignant [8]. This justifies the presenting symptoms in adults as either endocrinological or due to raised intracranial tension [4]. The prognosis for patients with adamantinomatous craniopharyngioma (ACP) is variable and depends on several factors, including the extent of surgical resection, tumor location, and involvement of critical structures such as the hypothalamus [4].

Other sellar lesions such as Rathk’s cleft cyst, differ from the craniopharyngioma histologically as there is evidence of squamous metaplasia with mucin-containing cells and there is the absence of wet keratin as well as calcification. Meningiomas usually present with a dural tail sign on contrast-enhanced magnetic resonance imaging (MRI). The presence of calcifications on imaging is crucial for differentiating other sellar masses and identifying small postoperative remnants that MRI cannot detect [2].

We present a case study of a 50-year-old female with a case of an adamantinomatous craniopharyngioma in the sellar, parasellar, and suprasellar regions.

## Case presentation

A 50-year-old female patient, with a BMI of 27 kg/m^2 ^presented at the neurology department with non-specific complaints of headache, generalized weakness, and occasional low-grade fevers for the last eight days. She had no complaints of vision, nausea, vomiting, trauma, or loss of consciousness. She reported no history of diagnosed co-morbidities or significant prior medical and surgical history. Her physical examination was unremarkable, and her vitals were normal. She had attained menopause at age 49. All lab investigations were done, and all the parameters were standard (Table 1).

**Table 1:** Laboratory results and reference ranges. Hb: hemoglobin; PT: prothrombin time; APTT: activated partial thromboplastin time; T3: triiodothyronine; T4: thyroxine; TSH: thyroid-stimulating hormone; LH: luteinizing hormone

Investigation	Observed value	Reference range
Hb %	10.1	Male 13-17 g/dL; Female 12-15 g/dL
RBC Count	4.68	Male 4.5-5.5 million/μL; Female 3.8-4.8 million/μL
WBC Count	19400	Male and female 4000-10000/μL
Total Platelet Count	1.57	Male and female 150000-400000(IU/μL)
PT	12	11-13.5 seconds
APTT	45	25-38 seconds
Sodium	136	135-145 mEq/L
Potassium	4.1	3.5-5.2 mEq/L
Urea	21	7-17 mg/dl
Creatinine	0.8	0.7-1.3 mg/dL
T3	0.584	0.970-1.69 ng/ml
T4	5.85	5.53-11.0 ng/dl
TSH	0.461	0.465-4.68 μIU/ml
LH	40.5	27.3-96.9 μIU/ml

**Figure 1:**
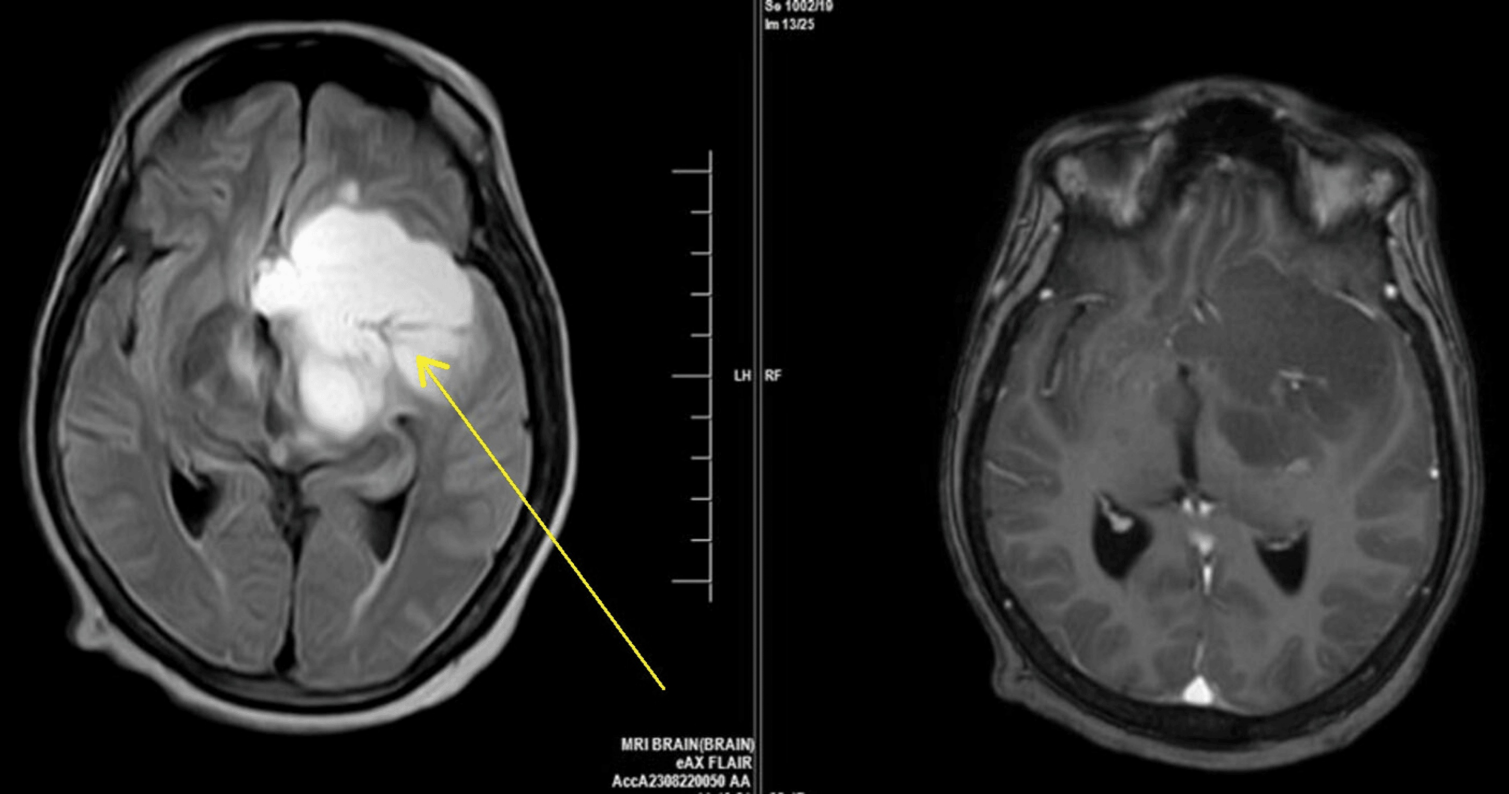
MRI Brain suggestive of lesion compressing the interpeduncular and left temporal regions (Yellow arrow).

The patient underwent a brain MRI with contrast and was found to have an extra-axial, non-enhancing, altered signal intensity mass lesion occupying the sellar, suprasellar, and parasellar regions. The mass was approximately 5.6 x 5.2 x 3.7 cm, causing effacement of the suprasellar cistern and appearing to compress the optic chiasma, hypothalamus, and thalamus. The lesion was superiorly found to extend into the left temporal region and posteriorly into the interpeduncular region, compressing the respective areas (Figure 1).

**Figure 2:**
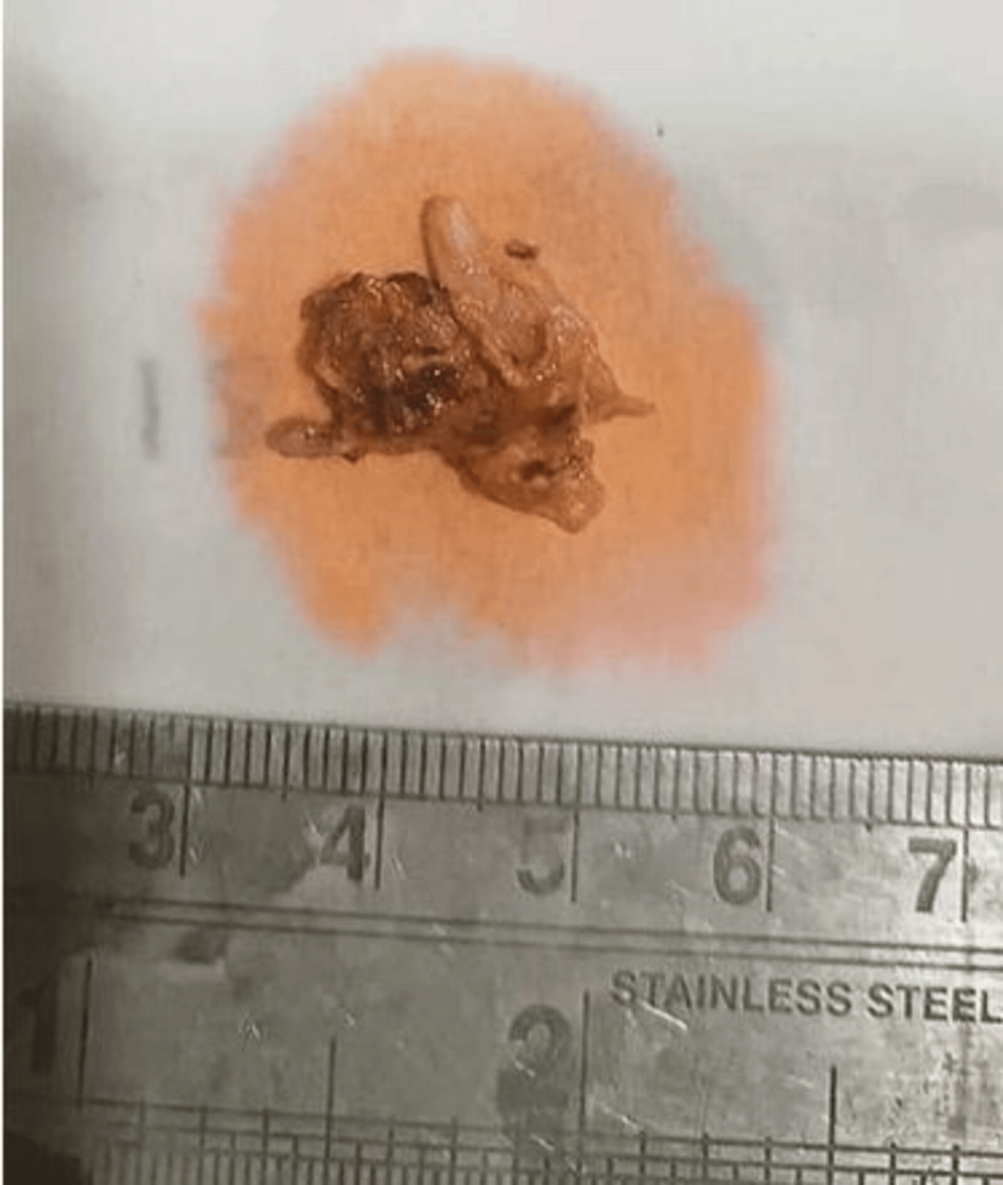
Gross image of the resected tumor (measuring 2 x 2 x 1 cm).

A left pterional craniotomy was performed, the Sylvian fissure was opened laterally, and the soft vascular wall of the cyst was opened. There was a release of opaque greenish oily fluid from the tumour, which smelled non-foul. The wall of the lesion was thick and adherent to the normal surrounding parenchyma, and upon excision, flakes were adherent to the vasculature. The mass was sent for histopathological examination. Grossly, we received multiple greyish-yellow tissue pieces measuring approximately 2 x 2 x 1 cm with areas resembling "dark motor oil" areas (Figure 2).

**Figure 3:**
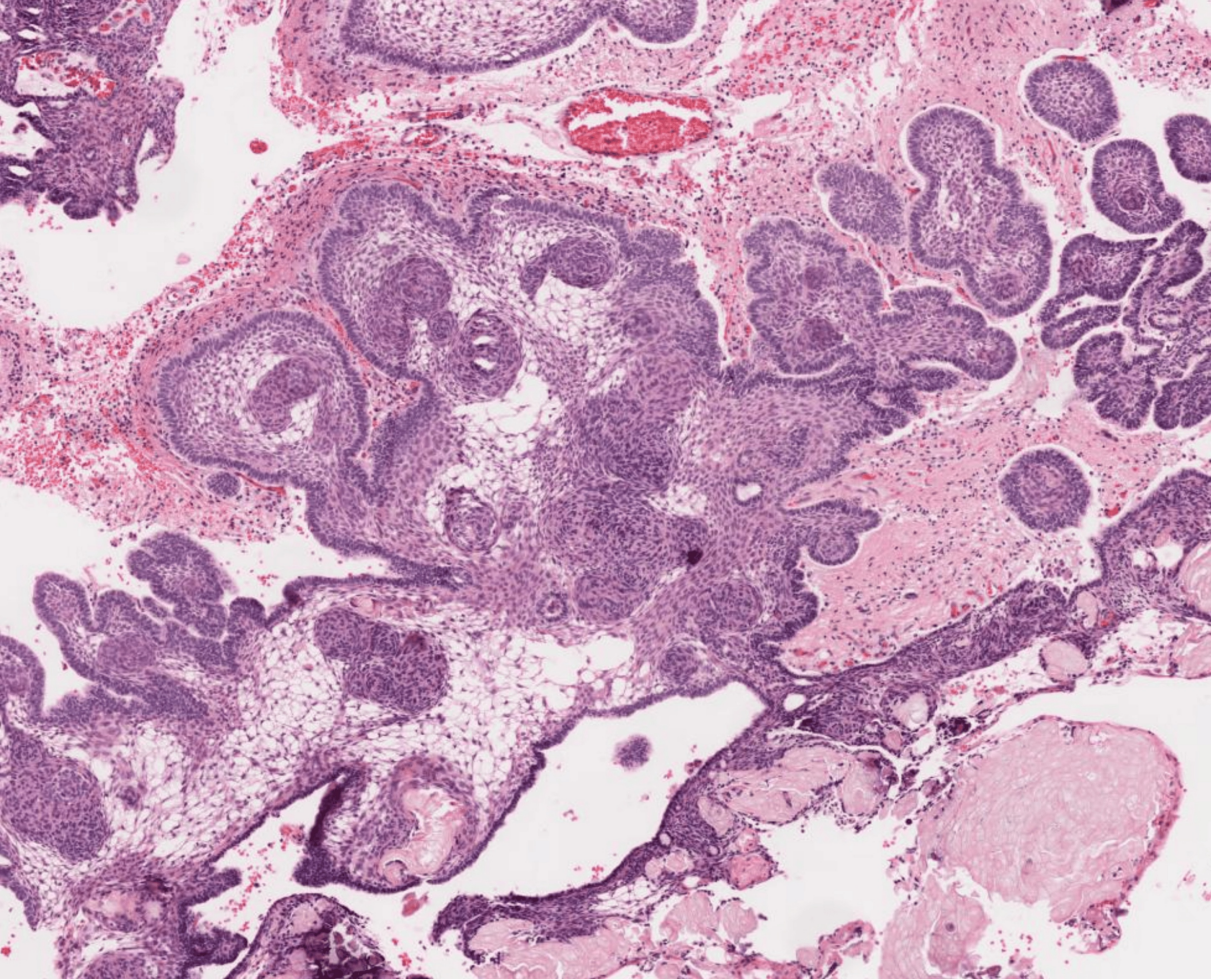
H&E 4x section shows both cystic and solid elements. H&E: hematoxylin and eosin stain

**Figure 4:**
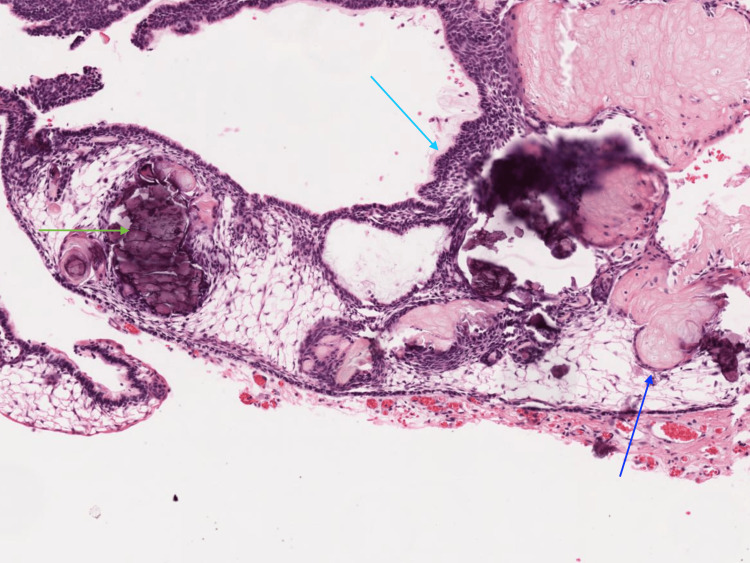
H&E 10x magnification, section shows a basal layer with basal palisading (light blue arrow); wet keratin (dark blue arrow); and a few areas of calcification (green arrow). H&E: hematoxylin and eosin stain

The tumor was diagnosed as an adamantinomatous craniopharyngioma on histopathological examination with both cystic and solid elements on 4x magnification (Figure 3). On 10x magnification, the section shows a basal layer with basal palisading (light blue arrow). There is evidence of wet keratin (dark blue arrow) and a few areas of calcification (green arrow) (Figure 4). All these histopathological features are suggestive of the diagnosis.

Preoperative medications given to the patient in the hospital are shown in Table 2.

**Table 2:** Treatment in the hospital BD – Twice a day, TDS- Thrice a day, IV- Intravenous, NS - normal saline

Medicine	Quantity	Dose	Days
Sodium chloride 0.9 % w/v (NS) IV	1	BD	1
Injection Ceftriaxone 1 gm IV	1	BD	1
Injection Pantaprazole 40 mg IV	1	TDS	1
Injection Tetanus Toxoid 0.5 ml	1	BD	1

No operative or postoperative complications were observed. The patient was stable and a prescription was given to the patient as shown in Table 3. The patient was discharged seven days postoperation with no complaints and was lost to follow-up.

**Table 3:** Prescription on discharge OD- Once a day, BD- Twice a day, TDS- Thrice a day, SOS- When it is needed

Medicine	Quantity	Dose	Days
Multivitamin and Inositol soft gelatin capsules	10	OD	10
Tab Metronidazol 400 mg	15	TDS	5
Tab Paracetamol 650 mg	10	SOS	SOS
Tab Ondansetron 4 mg	20	BD	10
Tab Levetiracetam 500 mg	10	BD	10
Tab Pantoprazole 40 mg	10	OD	10

## Discussion

This case report highlights a unique presentation of adamantinomatous craniopharyngioma in a 50-year-old female with an MRI shows the sellar, parasellar, and suprasellar regions, causing effacement of the suprasellar cistern and appearing to compress the optic chiasma, hypothalamus, and thalamus. Histopathological findings confirmed the diagnosis of adamantinomatous craniopharyngioma.

Craniopharyngiomas are rare brain tumors of the sellar and parasellar regions. The adamantinomatous subtype is more common in children, though it is frequently found in the adult population [9]. The presenting symptoms in adults are due to endocrinological disturbances in the hypothalamus-pituitary axis or raised intracranial tension due to a space-occupying lesion. The former may include sexual dysfunction with reduced follicle-stimulating hormone (FSH), luteinizing hormone (LH), adrenocorticotropic hormone (ACTH) deficiency, thyroid irregularities, or diabetes insipidus due to anti-diuretic hormone (ADH) deficiency [5,10,11]. The latter symptoms include headaches, nausea, vomiting, and visual changes often due to the compression of the optic chiasm which is anatomically below the sellar origin [1].

These are usually the first presenting symptoms in children, along with growth hormone deficiency [2]. The diagnosis of craniopharyngiomas is initiated by radiological imaging, with magnetic resonance imaging (MRI) proving to be the mainstay [4]. There appear to be solid and cystic components with calcification and fluid content contributing to the heterogeneous appearance. Computed tomography imaging often identifies microcalcifications compared to a popcorn-like or eggshell pattern [4]. All these findings allow craniopharyngiomas to be differentiated from other tumors in that region. A common rule, the 90% rule, has described the features of adamantinomatous craniopharyngioma (ACP). The rule claims that 90% of tumors are primarily cystic, 90% show calcifications, and 90% have walls that take up contrast. Further, radiological imaging helps note surrounding structures and the potential risks of long-term sequelae and surgical management. Histopathologically, adamantinomatous and papillary craniopharyngiomas are well distinguished, with adamantinomatous craniopharyngioma (ACP) showing palisading whorls and well-differentiated epithelium and papillary craniopharyngioma (PCP) showing projections with non-keratinising squamous epithelium and containing loosely structured connective tissue [4,12]. ACP often contains dark lipid-rich fluid, called "machine oil", within the cysts [12].

The management of craniopharyngioma, like most neoplasms, includes surgery (transcranial), radiation, a combination of the two, or chemotherapy [7]. While controversial in the pediatric population, surgical management is effective in reducing symptom burden in adults [10]. Both radical and limited approaches are performed, often combined with radiotherapy. Intracystic approaches with radiotherapy and chemotherapy like bleomycin are practical but associated with higher risks of neurotoxicity and death. In low-income countries with a rural population that is frequently lost to follow-up, complete surgical resection is a better option [5]. Radiation therapy is used for patients with residual disease or to prevent recurrences. Its goal is to reduce tumor burden while preserving essential neurological structures. Multiple studies have shown that radiation treatment decreases mortality and slightly reduces morbidity. Despite this, it has not been proven to lower the recurrence rate. Therefore, it remains an adjuvant method to neurosurgical intervention [5].

Reoccurrence of the tumor has been documented but the most common follow-up symptoms are endocrinological such as hypothyroidism, adrenal insufficiency, and diabetes insipidus due to hypothalamic insufficiency. Hypothalamic obesity is seen due to a lack of regulation of satiety and is often unresponsive to lifestyle modifications [2,13,14]. A documented spill from operative maneuvers into the spinal cord has been detected and resembles malignant dissemination [4]. Malignant transformation of ACP is extremely uncommon; increased expression of proliferation markers and TP53 have been shown in malignantly transformed craniopharyngioma (CP) tumors [15]. Numerous studies have evaluated the genetic alterations in craniopharyngiomas that could be crucial for future treatment strategies. β-catenin gene mutations have been documented in over 70% of adamantinomatous craniopharyngiomas (ACPs). With these sequelae in mind, urgent management and adequate follow-up are necessary for ideal patient care [16]. 

## Conclusions

This case report documents an atypical presentation of adamantinomatous craniopharyngioma in a 50-year-old female patient. Radiological studies confirmed a mass and histopathological findings showed basal palisading, wet keratin, and a few areas of calcification, which confirmed adamantinomatous craniopharyngioma. Our study suggested that nurses with specialized training in critical care, operating rooms, and perianesthesia are needed to provide care. This adamantinomatous craniopharyngioma should be handled cautiously, and tumors should be removed surgically. Adamantinomatous craniopharyngioma presents a difficulty for neurosurgeons due to the tumor's capacity to adhere to surrounding surfaces and the possibility of long-term neurological and physical problems before and after treatment. We have highlighted the significance of identifying calcifications and understanding the genetic mutations, such as β-catenin, that play a crucial role in the pathogenesis and future treatment strategies for ACP. The case underscores the importance of a multidisciplinary approach, integrating histopathology, imaging, and clinical findings to achieve an accurate diagnosis and optimal patient management. Our findings contribute to the existing body of knowledge, aiding clinicians and pathologists in differentiating ACP from other sellar/parasellar masses and guiding appropriate therapeutic interventions. 
